# Therapeutic response and survival time of immunobiologicals in patients with moderate to severe psoriasis^☆^

**DOI:** 10.1016/j.abd.2021.03.008

**Published:** 2021-11-20

**Authors:** Cynthia Cristina Ferreira Mota, Ricardo Romiti, Marcelo Arnone, Andre Luís da Silva Hirayama, Maria Denise Fonseca Takahashi

**Affiliations:** a Specialty Outpatient Clinic, Prefeitura Municipal de Santos, Santos, SP, Brazil; b Psoriasis Outpatient Clinic, Prefeitura Municipal de Santos, Santos, SP, Brazil; c Psoriasis Outpatient Clinic, Hospital das Clínicas da Universidade de São Paulo, São Paulo, SP, Brazil

Dear Editor,

Psoriasis is an inflammatory, chronic and recurrent disease with evident genetic influence. The intensity, extension and different manifestations associated with the disease guide therapeutic decisions. Advances in the knowledge of the disease immunopathology over the last decades have culminated in the development of new medications, called immunobiologicals, which act in a specific and precise way at different levels of the inflammatory cascade of psoriasis.^1^ With the introduction of anti-tumor necrosis factor-alpha (anti-TNF-α) drugs: etanercept (ETA), infliximab (INF), adalimumab (ADA), and certolizumab pegol (CP), followed by inhibitors of interleukin-12/23, ustekinumab (UST); inhibitors of interleukin 17: secukinumab (SEC) and Ixekizumab (IXE), and more recently inhibitors of interleukin 23 alone: ​​ Guselkumab (GUS) and Risankizumab (RISA), it has become possible to effectively treat severe and refractory forms of the disease, associated with a satisfactory safety profile. On the other hand, uncertainty regarding the choice of the most appropriate drug, the long-term sustained response, and the possibility of interrupting therapy has an impact on the therapeutic decision.

Drug survival is defined as the time from the beginning to the discontinuation of a certain treatment. The time interval from the start of the treatment to its discontinuation, as well as the reasons for this outcome, whether due to loss of efficacy, complications, or treatment abandonment, may vary in different populations with psoriasis.^2^, ^3^ To date, there are no data associating the therapeutic response to the survival time of immunobiologicals in patients with psoriasis in Brazil.

Aiming to determine the time of drug survival, a total of 229 treatments with immunobiological drugs were evaluated in 110 patients with moderate to severe psoriasis at Hospital das Clínicas, Universidade de São Paulo, in the state of São Paulo, Brazil, for a period of two years and analysed regarding the response to immunobiologicals, number of previous treatments and reason for discontinuation. The analysis of medical records also allowed the collection of data in relation to previous treatments since the introduction of immunobiologicals as a therapeutic option. Drug survival was defined as the time from the start of the treatment with the immunobiological, that is, the first dose until the occurrence of the event of interest (temporary/definitive discontinuation of treatment). Kaplan-Meier curves were used to estimate each of the drug survival probabilities and the difference between the drug survival curves was verified using the logrank test.

The comparative analysis between the five immunobiologicals showed that SEC was the drug with the longest time of survival, including 22 treatments (9.6%), followed by UST with 64 treatments (28%), ADA with 52 treatments (22.7%), ETA with 56 treatments (24.4%), and INF with 35 treatments (15.3%) (Fig. 1). Second-line cases showed lower drug survival compared to first-line cases, whereas third-line cases had lower survival compared to second-line cases, and so forth. Isolated analyses regarding the first and second-line drugs depicted Kaplan-Meier curves showing that UST had a higher probability of drug survival, with fewer failures. INF showed a lower probability of drug survival in two years, with more failures. These differences were shown to be statistically significant (Fig. 2).

**Figure 1:**
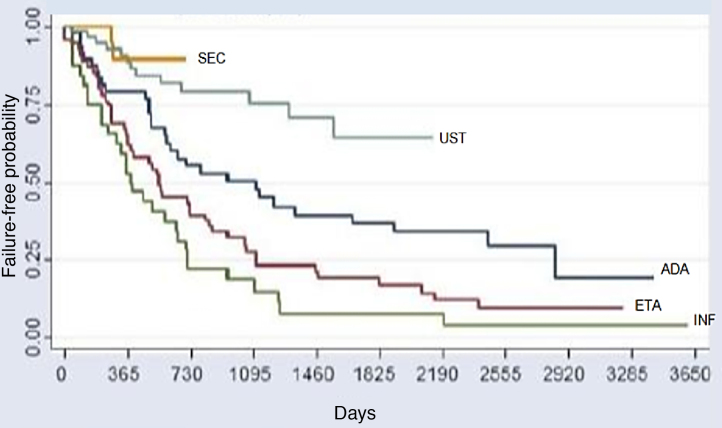
Kaplan-Meier curve for all treatments (p < 0.001). This chart reflects a better performance of secukinumab, followed by UST (second place), ADA (third place), ETA (fourth place), and INF (fifth place) over the period of 2 years (730 days). Through the analysis of the medical records, it was possible to assess the survival of some immunobiologicals for a period of up to 10 years (3,650 days).

**Figure 2:**
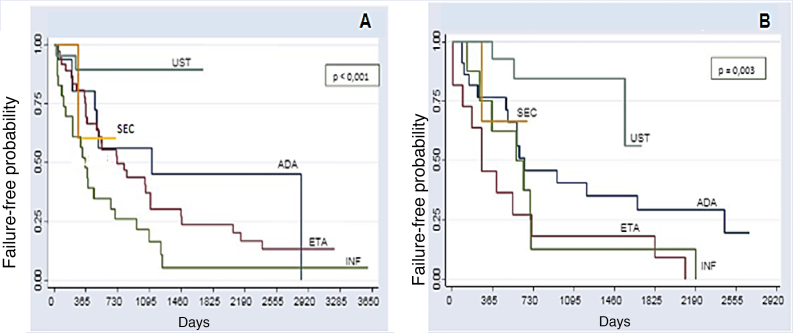
Kaplan-Meier curves for the treatments. (A), first-line (naïve) (p < 0.001); (B), Second-line (p = 0.003) in two years (730 days). In the first- and second-line groups, UST shows a higher probability of drug survival in two years; 89.2% and 83.3% respectively. INF shows a lower probability of drug survival in two years in the first and second-line groups (26.1% and 12.5%, respectively), with more failures. Through the analysis of the medical records, it was possible to assess the survival of some immunobiologicals for a period of up to 10 years (3,650 days).

Among the factors that led to treatment interruption, present in 95 of a total of 229 treatments (41.5%), the most frequent cause of temporary interruption was the lack of medication supply, observed in 76 of the analyzed cases (33.2%) (Table 1), whereas among the causes of definitive interruption, observed in 123 of a total of 229 treatments (49.6%), the main cause was a secondary treatment failure, observed in 26.6% of the analyzed treatments (Table 2). Overall, the primary failure was considered when the patient did not show a PASI50 response after 24 weeks of treatment and occurred in 5.2% of cases. When analyzing the results, one should consider the small number of cases treated with SEC, a drug that was only approved in Brazil in 2016. Similarly, data from more recently approved immunobiologicals such as CP, IXE, GUS, and RISA are not included in the results of this study.

**Table 1:** Reasons for temporary interruption of treatments.

	Reasons	n (%)
n = 229 Treatments	Failure of supply in SUS	76 (33.2)
	Others^a^	9 (3.9)
	Pregnancy	3 (1.3)
	Lost to follow-up	3 (1.3)
	Surgery	2 (0.9)
	Repeated UAIs	2 (0.9)
Total		95 (41.5)

SUS, *Sistema Único de Saúde*; UAIs, Upper Airway Infections.

a Others: Nonspecific symptoms such as headache, myalgia, arthralgia, nausea.

**Table 2:** Reasons for definitive interruption of treatments.

	Reasons	n (%)
n = 229 Treatments	Secondary failure	61 (26,6)
	Others	19 (8,3)
	Primary failure	12 (5,2)
	Failure of supply in SUS	12 (5,2)
	Tuberculosis	8 (3,5)
	Pregnancy	4 (1,7)
	Protocol	2 (0,9)
	Stomach cancer	1 (0,4)
	Hernia surgery	1 (0,4)
	Hepatotoxicity	1 (0,4)
	UAI	1 (0.4)
	Infusion reaction	1 (0.4)
Total		123 (53.7)

Others, Headache, nausea, myalgia, and arthralgia; SUS, Unified Health System; Protocol, Patients who used infliximab only in the induction phase to control erythrodermic psoriasis; UAI, Upper Airway Infection.

According to the literature, one of the main causes for the discontinuation of an immunobiological is the loss of efficacy, also called secondary failure.^4^, ^5^ Our results demonstrate in an unprecedented way that the medication supply failure was the main reason for the temporary interruption of treatments. It is noteworthy that, when resuming the immunobiological treatment after a prolonged interruption, the chance of a lower response should always be considered.^6^, ^7^ These facts reinforce the importance and need for continuous and regular provision of these medications by the providers.

The occurrence of tuberculosis leading to treatment discontinuation was exclusive to anti-TNFs, as well as the few cases of infusion reaction, UAIs, hepatotoxicity, and cancer. The lack of medication in the Brazilian Unified Health System (SUS, *Sistema Único de Saúde*), leading to the definitive interruption of the therapy, was more frequent with UST and SEC. Regarding the main cause of discontinuation, secondary failure occurred with all immunobiologicals, with a variation only in the time of its occurrence.

The results shown here provide a regional analysis of the time of survival of immunobiological drugs and the main reasons for treatment discontinuation in a public institution, which can help in the planning and monitoring of treatments with immunobiologicals, both by the prescribing physicians and the health managers.

## Financial support

None declared.

## Authors’ contributions

Cynthia Cristina Ferreira Mota: Terminology, conceptualization, methodology, investigation, resources, data curation, writing-original draft, visualization, project administration.

Ricardo Romiti: Methodology, investigation, writing review and editing, supervision.

Marcelo Arnone: Investigation.

Andre Luís da Silva Hirayama: Investigation.

Maria Denise Fonseca Takahashi: Investigation.

## Conflicts of interest

None declared.
